# Multiple mesenteric lymphadenopathies in pediatric with ulcerative colitis: A case report

**DOI:** 10.1016/j.radcr.2023.10.046

**Published:** 2023-11-24

**Authors:** Saumy Dewi Ratih, Firdian Makrufardi, Annisa Fairuz Nur Azizah, Wahyu Damayanti

**Affiliations:** Department of Child Health, Faculty of Medicine, Public Health and Nursing, Universitas Gadjah Mada/Dr. Sardjito Hospital, Yogyakarta, Indonesia

**Keywords:** Ulcerative colitis, Pediatric, Lymphadenopathy, Case report

## Abstract

Ulcerative colitis (UC) is one of the 2 major disorders in pediatric inflammatory bowel disease (IBD). Differentiating IBD at an early stage remains difficult, and abdominal imaging and early precise investigations are crucial. A 2-year-old girl was referred to the emergency department after experiencing colicky abdominal pain for 1 month. She had bloody stool 4 days before admission with the frequency of about 1-2 times per day. She also experienced anorexia, nausea, and weight loss. From Abdominal CT-Scan with contrast, multiple mesenteric lymphadenopathies accompanied by liver enlargement and minimal ascites were found. A colonoscopy showed multiple ulcers in the rectum and sigmoid colon. The histology of the gastric and colon showed lymphocyte infiltration in lamina propria. Children with UC usually present with the classic symptoms of weight loss, abdominal pain, and bloody diarrhea. The UC patients could present also with nonclassic symptoms of poor growth, anemia, or extraintestinal manifestations. The presence of inflamed mesenteric lymph nodes in the inflammatory process in UC can be associated with peri-intestinal inflammatory reactions. Mesenteric lymphadenopathies can happen in UC and early investigations using colonoscopy and biopsy are important investigative procedures to evaluate patients with UC.

## Introduction

Ulcerative colitis (UC) is one of the 2 major disorders in inflammatory bowel disease (IBD), and its prevalence is increasing globally [1]. It is estimated that 17.6 per 100,000 (95% CI: 15.4-19.9) children have ulcerative colitis, and the prevalence will continue to rise year after year. UC in children is most common in teenagers, but it can occur at any age [2]. According to one study, the prevalence of UC was 20% in people under the age of 20, 4% in children under the age of 5, and 1% in infants [[3], [4]]. A previous study showed that the mesenteric lymph nodes were the most commonly associated lymph nodes in IBD [5]. Interestingly, mesenteric lymphadenopathy is more common in Crohn's disease patients [6]. However, new evidence suggests that mesenteric lymphadenopathy occurs in UC [5]. Differentiating IBD at an early stage remains difficult, and abdominal imaging and early precise investigations are important [7]. Here, we present a case of a 2-year-old girl who presented with UC and underwent timely investigations, including colonoscopy and biopsy, which assisted in the diagnosis. This case was reported in line with the CARE guidelines [8].

## Case presentation

A 2-year-old girl was referred to the emergency department after experiencing colicky abdominal pain for 1 month. She had bloody stool 4 days before admission with the frequency of about 1-2 times per day. She also experienced anorexia, nausea, and weight loss. There were no symptoms of fever, jaundice, mouth ulcers, or joint pains. She was treated several times by different doctors for these symptoms, but she did not improve. She had no history of food allergies.

Physical examination revealed that she was afebrile, anicteric, not dehydrated, and weighed 10 kg. There was no peripheral lymphadenopathy, desquamation, or discoloration of the skin. Mucous membranes and nails were both normal. We discovered severe periumbilical tenderness but no palpable abdominal mass, hepatomegaly, or splenomegaly. Initial testing revealed a hemoglobin of 10.6 g/dL, a total leukocyte count of 16,520/mm^3^, a neutrophil differential of 69.4%, and a lymphocyte differential of 22.7%. Platelets were counted at 2.6 × 109 cells/mm^3^. Urinalysis, renal function tests, and coagulation tests were normal. On fecal examination, there were brown liquid stools with mucus and bacteria, but no stool cultures were obtained. On stool microscopy, no eggs or intestinal parasites were found. The Mantoux test found a negative result.

**Fig. 1:**
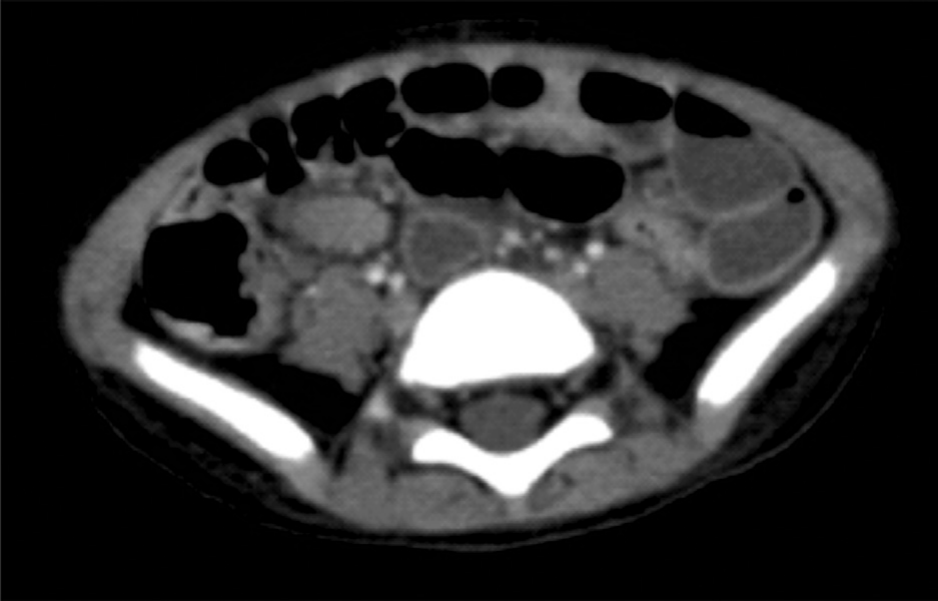
Abdominal CT-Scan with contrast showing multiple mesenteric lymphadenopathies accompanied by liver enlargement and minimal ascites.

**Fig. 2:**
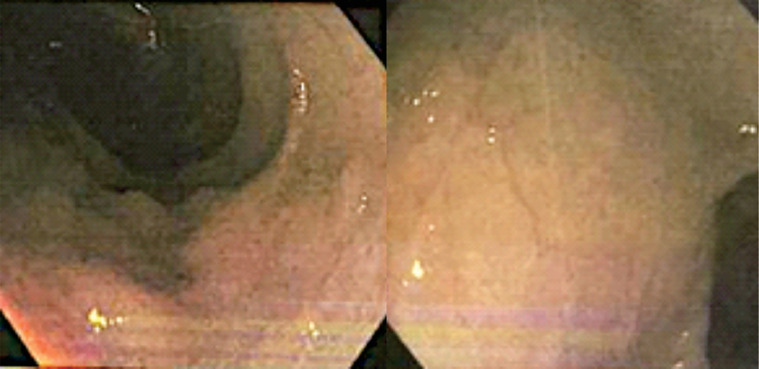
Colonoscopy showing multiple ulcers in rectum and sigmoid colon.

**Fig. 3:**
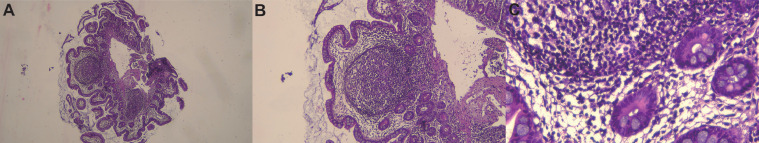
Histopathology of colon showed showing lymphocytes infiltration in lamina propria and basal lymphoplasmacytosis (A) 4x magnification, (B) 10x magnification, (C) 40x magnification.

Abdominal ultrasonography showed ascending colon wall thickening caused by a suspect inflammatory process and cystitis. From the Abdominal CT-Scan with contrast, multiple mesenteric lymphadenopathies accompanied by liver enlargement and minimal ascites were found (Fig. 1). Colonoscopy showed multiple ulcers in the rectum and sigmoid colon (Fig. 2). Caecum, ascending, transverse, descending, sigmoid colon, and rectum biopsy tissues showed active chronic colitis. Stomach and duodenal biopsy showed erosive gastroduodenitis. The histology of the gastric and colon showed lymphocyte infiltration in lamina propria (Fig. 3). Basal plasmacytosis or plasma cells extending below crypt endings in more than 2 foci and lymphoid follicle formation. Taken together, these findings highlight the suggestive findings of ulcerative colitis. Then, she was diagnosed with ulcerative colitis with a pediatric ulcerative colitis activity index (PUCAI) score of 30. The patient was given management based on algorithms of Indonesian Pediatric Society guidelines.

## Discussion

Here, we reported a case of a 2-year-old female patient with ulcerative colitis in our tertiary hospital. Children with UC usually present with the classic symptoms of weight loss, abdominal pain, and bloody diarrhea [9]. The UC patients could present also with nonclassic symptoms of poor growth, anemia, or extraintestinal manifestations [10]. The current research examined mesenteric lymph nodes in patients with UC, which is a chronic inflammatory disorder of the large intestine [5]. The presence of inflamed mesenteric lymph nodes in the inflammatory process in UC can be associated with peri-intestinal inflammatory reactions [5], [11].

In this study, our patient had a PUCAI score of 30 and was classified as having mild severity. The Pediatric Ulcerative Colitis Activity Index (PUCAI) is a clinical tool used to assess disease activity, which includes abdominal pain, rectal bleeding, stool consistency, number of stools per 24 hours, nocturnal stools, and activity level [12]. The PUCAI score is between 0 and 85, with higher scores indicating more severe disease activity. The PUCAI index is used in clinical practice to guide treatment decisions, including the initiation of medical therapy or escalation of therapy for children with ulcerative colitis [13].

Lymphoid tissue in the gastrointestinal tract varied according to fluid clearance, fat absorption, and immune cell clearance [14]. Chronic IBD inflammation affects the lymphatic system, though its effect on lymphatics is correlated with the inflamed part of the gastrointestinal tract, which is more prominent in the small intestine than in the colon [5], [15]. The lymphoid tissue of the colon is normally found in the muscularis mucosa layer and is absent from the rest of the mucosa [16]. Chronic UC inflammation causes submucosal lymphoid hyperplasia, lymphatic angiogenesis, and lymph node formation [5].

Neoplastic, inflammatory, and infectious processes are the most common causes of mesenteric lymphadenopathy [17]. Lymphadenopathy affects the prognosis of the disease, which in turn will affect further management [18]. A study showed that increased lymphangiogenesis was associated with a better prognosis of IBD, while increased lymphangiectasia, lymphadenopathy, and lymphatic vessel occlusion were associated with a worse prognosis [19]. IBD lymphatic system therapies aim to increase lymphangiogenesis by inducing lymphangiogenic factors and inhibiting their antagonists [20]. Furthermore, mesenteric lymphadenopathy could be the only sign of an underlying inflammatory or infectious process causing abdominal pain [17]. The distribution of lymph nodes may indicate the precise nature of the underlying disease process, allowing the appropriate treatment to be instituted [21].

Colonoscopy is the first-line investigative procedure in the diagnosis of patients with suspected IBD due to wide availability [22]. The therapeutic goal in UC is to achieve clinical and laboratory remission of the disease while allowing the patient to function as normally as possible [23]. Anti-inflammatory therapy with 5-aminosalicylic acid (5-ASA), such as sulfasalazine and mesalamine, is the mainstay of UC outpatient management [5]. Acute flares of UC in children respond well to corticosteroids, but the numerous side effects prevent long-term use [23]. Total colectomy is the choice of treatment for intractable or fulminant colitis with the major complication being pouchitis [24]. UC can compromise mucosal integrity, allowing bacteria to enter submucosal tissues and cause necrosis and peritonitis, which commonly responds to oral metronidazole or ciprofloxacin treatment [25]. Together, with a precise diagnostic procedure, the diagnosis of UC in children can be stated as early as possible.

## Conclusions

Mesenteric lymphadenopathies can occur in cases of UC, and early investigations involving colonoscopy and biopsy are essential procedures for evaluating patients with UC. Clinicians should deliver timely and personalized healthcare to enhance patient results.

## Patient consent

Written informed consent was obtained from the parent for publication of this case report and accompanying images. A copy of the written consent is available for review by the Editor-in-Chief of this journal on request.

## Provenance and peer review

Not commissioned, externally peer-reviewed.
